# Determinants of smoking initiation among women in five European countries: a cross-sectional survey

**DOI:** 10.1186/1471-2458-10-74

**Published:** 2010-02-17

**Authors:** Debora L Oh, Julia E Heck, Carolyn Dresler, Shane Allwright, Margaretha Haglund, Sara S Del Mazo, Eva Kralikova, Isabelle Stucker, Elizabeth Tamang, Ellen R Gritz, Mia Hashibe

**Affiliations:** 1 International Agency for Research on Cancer, Lyon, France; 2 Department of Epidemiology, University of California Los Angeles, School of Public Health, Los Angeles, California, USA; 3 Tobacco Prevention and Cessation Program, Arkansas Department of Health, Little Rock, Arkansas, USA; 4 Department of Public Health and Primary Care, Trinity College Dublin, Republic of Ireland; 5 Tobacco Control, National Institute of Public Health, Östersund, Sweden; 6 Health Professionals Against Tobacco, Stockholm, Sweden; 7 Institute of Hygiene and Epidemiology, First Faculty of Medicine, Charles University and General University Hospital, Prague, Czech Republic; 8 3rd Medical Department - Department of Endocrinology and Metabolism, First Faculty of Medicine, Charles University and General University Hospital, Prague, Czech Republic; 9 INSERM U 754 - IFR69, Villejuif, France; 10 Regione del Veneto - Direzione Prevenzione, Servizio di Sanità Pubblica e Screening, Venezia, Italy; 11 Department of Behavioral Science, The University of Texas MD Anderson Cancer Center, Houston, Texas, USA; 12 Division of Public Health, Department of Family & Preventive Medicine, University of Utah School of Medicine, Salt Lake City, Utah, USA

## Abstract

**Background:**

The rate of smoking and lung cancer among women is rising in Europe. The primary aim of this study was to determine why women begin smoking in five different European countries at different stages of the tobacco epidemic and to determine if smoking is associated with certain characteristics and/or beliefs about smoking.

**Methods:**

A cross-sectional telephone survey on knowledge and beliefs about tobacco was conducted as part of the Women in Europe Against Lung Cancer and Smoking (WELAS) Project. A total of 5 000 adult women from France, Ireland, Italy, Czech Republic, and Sweden were interviewed, with 1 000 from each participating country. All participants were asked questions about demographics, knowledge and beliefs about smoking, and their tobacco use background. Current and former smokers also were asked questions about smoking initiation. Basic statistics on the cross-sectional data was reported with chi-squared and ANOVA p-values. Logistic regression was used to analyze ever versus never smokers. Linear regression analyses were used to analyze age of smoking initiation.

**Results:**

Being older, being divorced, having friends/family who smoke, and having parents who smoke were all significantly associated with ever smoking, though the strength of the associations varied by country. The most frequently reported reason for initiation smoking was friend smoking, with 62.3% of ever smokers reporting friends as one of the reasons why they began smoking. Mean age of smoking initiation was 18.2 years and over 80% of participants started smoking by the age of 20. The highest levels of young initiators were in Sweden with 29.3% of women initiating smoking at age 14-15 and 12.0% initiating smoking younger than age 14. The lowest level of young initiators was in the Czech Republic with 13.7% of women initiating smoking at age 14-15 and 1.4% of women initiating smoking younger than age 14. Women who started smoking because their friends smoked or to look 'cool' were more likely to start smoking at a younger age. Women who started smoking to manage stress or to feel less depressed were more likely to start smoking at an older age.

**Conclusions:**

In all five participating countries, friends were the primary factor influencing ever smoking, especially among younger women. The majority of participants began smoking in adolescence and the average reported age of smoking initiation was youngest in Sweden and oldest in the Czech Republic.

## Background

The prevalence of smoking among women varies greatly across European countries, ranging from less than 10% to over 30% [1]. Overall, however, Europe has some of the highest levels of female smoking in the world [2]. Furthermore, while smoking among men has declined in some European countries, it is still increasing or stabilised among women in most European countries [3-5].

Reasons for smoking initiation differ across cultures but certain factors have an established association with smoking. Parental, sibling, and friend smoking all have been shown to be strongly associated with an individual's smoking status [6-13]. Low academic achievement as well as low socioeconomic status are also associated with smoking status [8,12,14-16]. Women, just like men, begin to smoke in increasing proportions in the population because of marketing [17] and the tobacco industry significantly targets women with focused advertisements and promotions [18]. Women appear to have different reasons for smoking than men; for example, tension reduction and stimulation are thought to be more strongly associated with women smoking than with men [19,20]. There is also evidence that weight concerns play a role in the decision to smoke among women [21-24].

Over 60% of lung cancer cases in European women are attributed to their smoking history [25,26]. Thus with smoking rates in women rising, it is not surprising that lung cancer rates in women have also increased in most European countries [27,28]. In order to prevent further increases in smoking-related mortality, it is important that anti-tobacco measures target women in Europe.

Many European countries have recently made substantial changes in policy toward smoking and tobacco. The European Community as an entity, in addition to all major European Region countries, except the Czech Republic, has ratified the World Health Organization's Framework Convention on Tobacco Control (FCTC). The FCTC became international law in 2004 and is a comprehensive tobacco control treaty which includes an article for 100% smoke-free policies. In the same year, Ireland implemented a nation-wide ban on smoking in bars and restaurants. Since then, other European countries, including France, Italy and Sweden, have followed with similar national smoke-free legislation. Other measures against tobacco include increasing taxes on tobacco, improving consumer information, health warning labels on tobacco products, banning tobacco advertising promotion and sponsorship, and facilitating access to smoking cessation treatment [29].

The objective of this study is to determine if ever smoking and smoking initiation are associated with certain characteristics or beliefs about smoking in women in five European countries. In particular, this study aims to elucidate if these characteristics or beliefs about smoking are different for women in countries at different stages in the tobacco epidemic, with northern European countries generally being at a more advanced stage of the epidemic than southern European countries. As current policies are being rapidly reformed in Europe, and as rates of smoking are rising among European women, understanding reasons why European women smoke is crucial in guiding future tobacco control measures.

## Methods

### Participants

As part of the Women in Europe Against Lung Cancer and Smoking (WELAS) Project, supported by the European Commission, a cross-sectional land-line telephone survey on health attitudes and knowledge was conducted from June to July 2008 in five European countries: France, Ireland, Italy, Czech Republic, and Sweden [30]. These countries were chosen because they are at different stages of the tobacco epidemic and at different stages of enacting tobacco control measures. This study was approved by the International Agency for Research on Cancer ethical review committee.

In total, 5 000 women participated in this survey, with 1 000 women from each participating country. Of the women reached who were eligible for participation, response rates were 64.8% in France, 41.4% in Italy, 59.0% in Sweden, 54.6% in Ireland, and 30.6% in the Czech Republic.

### Sampling

The goal was to have a sample of women from each country that was nationally representative with regard to age and smoking status. Our sampling frame included all adult women 18 years of age and older who had a listed telephone number in the five participating countries. When a household was reached, the woman interviewed was selected at random from all adult women living in that household. To ensure adequate power to examine subsamples of smokers and nonsmokers for each question, smokers were oversampled to reach 28% of subjects; weighting was done in the analyses to make results representative of each country's female population.

### Measures

The survey was identical across countries, with attention given to translation issues. Surveys were translated by native speakers from each participating country and translations were checked by another native speaker in each country. All participants were asked questions about demographics, beliefs about smoking, and tobacco use background in the language native to each country. Current and former smokers were also asked questions about starting smoking.

Our primary outcomes of interest were 1) ever smoking and 2) age at smoking initiation. Women who reported smoking "every day", "some days or occasionally", or "not anymore (>100 cigarettes in life)", were combined to create a group of "ever smokers". Participants with a smoking history were asked at what age they first started smoking regularly.

Background characteristics with prior associations with smoking initiation were included in the analyses, such as age, education, marital status, and income. Since educational systems differ across countries, we measured education as the age at last year of education. Age at last year of education was categorized into less than 16, 16 to 19, 20 to 25, and over 26 years old. These categories were meant to approximately reflect those who did not finish secondary school, those who finished secondary school, those who went to university, and those who had post-graduate education. Respondents reporting being divorced and separated were combined into one category for analysis. In order to create comparable categories between countries, information on personal household income was collected relative to the national median household disposable income for 2005-6 for their respective country. For example, the actual question asked in Ireland was "Given that the median annual household income in Ireland is approximately 43 000 euro, would you say that your household income is well below, below, around, above or well above the median?" The national medians provided to respondents were: 30 000 euro for France (2005; source INSEE), 230 000 krona for Sweden (2006; source Statistiska centralbyran), 32 000 euro for Italy (2006; source Bank of Italy), 110 000 koruna for the Czech Republic (2006; source Czech Statistical Office), 43 000 euro for Ireland (2006; source Central Statistics Office Ireland).

Information on friends and family smoking was also collected for the analysis. Those who reported that "more than half" or "most or all" of their friends and family smoked were combined into one category for analyses. Women were also asked if any of their parents, step-parents, or guardians smoked.

Using an open-ended question format, ever smokers were asked to list reasons why they started smoking. Answers were recorded as the participant responded and participants could list more than one reason.

### Analyses

All analyses were done with SAS version 9. Chi-squared tests were conducted to test differences between countries. Logistic regression analyses were conducted to determine factors associated with ever smoking and young smoking initiation. Mean age at initiation was compared across country, age, income category, and education. When comparing age cohorts, those who initiated after the age of 24 (~10%) were excluded to remove age-related bias. One-way ANOVA tests were used to test differences in means. Linear regression was conducted to determine if any reasons for smoking initiation were associated with younger age at initiation.

## Results

A total of 5 000 women were interviewed for this study, including 1 000 women from each of the five countries. Demographic characteristics are listed in Table 1. Over 40% of participants (n = 2378) had ever smoked (>100 cigarettes in lifetime). Age ranged from 18 to 95 with approximately 37% of participants over the age of 55 (Table 1).

**Table 1:** Women in Europe Against Lung Cancer and Smoking (WELAS) Project demographics by country, weighted.

	All		France	Ireland	Italy	Czech Republic	Sweden	χ^2^
	(n = 5000)		(n = 1000)	(n = 1000)	(n = 1000)	(n = 1000)	(n = 1000)	p-value
	n	%	%	%	%	%	%	(df = 4)
**Ever smoker**								
*(>100 cigarettes in life)*	2622	41.3	37.7	49.0	39.7	34.3	45.8	<0.0001
**Current smoker**	1421	20.5	19.9	23.5	17.5	19.2	18.6	0.0094

**Age**								
*18-24*	426	8.5	8.2	11.4	6.5	8.3	8.2	<0.0001
*25-34*	939	18.8	17.2	22.0	19.4	20.1	15.2	
*35-44*	916	18.3	18.6	19.8	19.0	16.0	18.2	
*45-54*	883	17.7	18.1	16.8	16.9	17.9	18.6	
*55+*	1836	36.7	37.9	30.0	38.2	37.7	39.8	
**Age at last education**								
*<16*	601	12.5	5.0	13.2	25.2	6.0	12.3	<0.0001
*16-19*	2066	42.6	37.1	52.3	34.4	49.8	39.3	
*20-25*	1683	35.0	53.7	29.2	30.0	35.4	27.8	
*>25*	476	9.7	4.0	5.3	10.4	8.9	20.6	
*(missing)*	*(n = 174)*		*(n = 86)*	*(n = 27)*	*(n = 5)*	*(n = 13)*	*(n = 43)*	
**Marital Status**								
*Married*	1979	41.1	46.3	37.1	52.1	38.0	31.9	<0.0001
*Divorced/Separated*	727	14.2	20.5	8.2	7.8	16.9	17.5	
*Widowed*	605	12.8	3.5	18.2	11.6	22.1	8.9	
*Never married*	1074	21.6	11.2	33.0	25.4	21.6	16.6	
*Unmarried couple*	514	10.4	18.5	3.6	3.1	1.4	25.1	
*(missing)*	*(n = 101)*		*(n = 38)*	*(n = 28)*	*(n = 5)*	*(n = 22)*	*(n = 8)*	
**Income**								
*Well below the median*	463	9.2	7.5	7.7	17.5	8.2	5.2	<0.0001
*Below the median*	1183	23.5	24.1	25.3	29.7	19.2	19.2	
*Around the median*	1386	27.6	27.4	26.5	21.6	40.8	21.7	
*Above the median*	805	16.0	14.9	21.9	7.4	14.5	21.2	
*Well above the median*	165	3.3	2.8	3.5	0.5	0.8	9.1	
*Refused to answer*	998	20.4	23.3	15.1	23.31	16.5	23.6	
**Friends/family smoke**								
*None*	991	21.1	18.4	20.1	20.2	16.7	29.9	<0.0001
*Few*	2304	47.8	42.5	52.2	44.5	45.9	53.7	
*Less than half*	587	11.3	13.6	10.3	10.5	13.3	8.5	
*About half or more*	1094	19.9	25.5	17.4	24.8	24.1	8.0	
*(missing)*	*(n = 24)*		*(n = 1)*	*(n = 7)*	*(n = 9)*	*(n = 4)*	*(n = 3)*	
**Parents smoke**								
*No*	2101	43.1	41.4	36.2	43.2	50.9	43.8	<0.0001
*Yes*	2899	56.9	58.6	63.8	56.9	49.1	56.2	

Older age was significantly associated with ever smoking (Table 2). In particular, older women in Italy, the Czech Republic and Sweden all showed a significant increasing trend of a smoking history. Education and income measures were not significantly associated with smoking. In the combined analysis and in country-specific analyses for Ireland, Italy and Sweden, divorce or separation was significantly associated with ever smoking. In the Czech Republic, being widowed was significantly associated with not smoking, as compared to married women (Table 2). The widowed Czech women tended to be older, less educated and have a lower income (data not shown).

**Table 2:** Factors associated with ever smokers compared to never smokers by country, weighted.

	All (n = 5000)	France (n = 1000)	Ireland (n = 1000)	Italy (n = 1000)	Czech Republic (n = 1000)	Sweden (n = 1000)
	**OR (95% CI)**	**OR (95% CI)**	**OR (95% CI)**	**OR (95% CI)**	**OR (95% CI)**	**OR (95% CI)**

**Age**						
*18-24*	1.00	1.00	1.00	1.00	1.00	1.00
*25-34*	1.43 (1.10, 1.86)	2.02 (1.08,3.78)	1.66 (1.01, 2.75)	1.44 (0.73, 2.83)	0.82 (0.44, 1.55)	1.56 (0.81, 3.01)
*35-44*	1.58 (1.20, 2.08)	1.33 (0.71, 2.49)	1.81 (1.04, 3.15)	1.82 (0.90, 3.67)	1.11 (0.54, 2.29)	2.50 (1.29, 4.83)
*45-54*	1.77 (1.33, 2.34)	1.53 (0.83, 2.92)	1.70 (0.95, 3.05)	2.66 (1.30, 5.45)	1.37 (0.66, 2.86)	2.57 (1.33, 4.94)
*55+*	1.68 (1.28, 2.20)	1.53 (0.83, 2.79)	1.69 (0.94, 3.04)	1.98 (0.99, 3.93)	1.67 (0.81, 3.46)	2.61 (1.37, 4.96)
**Age at last education**						
*<16*	0.98 (0.75, 1.26)	0.56 (0.25, 1.26)	1.16 (0.57, 2.37)	0.89 (0.52, 1.52)	0.86 (0.39, 1.90)	0.96 (0.59, 1.55)
*16-19*	1.09 (0.88, 1.36)	0.63 (0.30, 1.33)	1.00 (0.54, 1.87)	1.13 (0.69, 1.83)	1.23 (0.72, 2.08)	1.34 (0.92, 1.96)
*20-25*	0.85 (0.68, 1.07)	0.55 (0.26, 1.13)	0.66 (0.35, 1.25)	0.96 (0.59, 1.57)	0.91 (0.52, 1.58)	0.83 (0.56, 1.24)
*>25*	1.00	1.00	1.00	1.00	1.00	1.00
**Marital status**						
*Married*	1.00	1.00	1.00	1.00	1.00	1.00
*Divorced/Separated*	1.49 (1.23, 1.80)	1.16 (0.79, 1.67)	1.91 (1.12, 3.25)	2.00 (1.20, 3.35)	1.42 (0.94, 2.16)	1.73 (1.14, 2.63)
*Widowed*	0.83 (0.67, 1.04)	1.44 (0.67, 3.10)	0.77 (0.49, 1.20)	0.89 (0.54, 1.46)	0.53 (0.33, 0.84)	1.36 (0.79, 2.36)
*Never married*	1.13 (0.94, 1.35)	1.86 (1.14, 3.03)	1.28 (0.90, 1.85)	0.97 (0.66, 1.41)	1.21 (0.75, 1.94)	0.89 (0.56, 1.39)
*Unmarried couple*	1.26 (1.01, 1.58)	1.22 (0.82, 1.82)	1.85 (0.85, 4.02)	1.66 (0.76, 3.60)	1.64 (0.48, 5.59)	1.20 (0.83, 1.73)
**Income**						
*Well below the median*	1.27 (1.00, 1.61)	1.57 (0.90, 2.74)	1.45 (0.83, 2.54)	1.11 (0.71, 1.75)	0.73 (0.40, 1.34)	1.87 (0.93, 3.75)
*Below the median* *Around the median*	1.12 (0.94, 1.33) 1.00	1.17 (0.79, 1.74) 1.00	1.16 (0.80, 1.69) 1.00	0.98 (0.67, 1.43) 1.00	1.23 (0.81, 1.86) 1.00	1.03 (0.67, 1.59) 1.00
*Above the median*	1.17 (0.97, 1.41)	1.61 (1.05, 2.49)	1.03 (0.69, 1.53)	1.00 (0.57, 1.76)	1.16 (0.76, 1.79)	1.13 (0.75, 1.72)
*Well above the median*	0.97 (0.68, 1.36)	0.85 (0.36, 2.01)	0.47 (0.21, 1.04)	2.13 (0.23, 20.14)	2.14 (0.42, 10.79)	1.42 (0.84, 2.43)
*Refused to answer*	0.87 (0.72, 1.04)	1.14 (0.75, 1.74)	0.88 (0.57, 1.35)	0.56 (0.37, 0.85)	0.81 (0.52, 1.25)	0.90 (0.60, 1.34)
**Friends/family smoke**						
*None*	1.00	1.00	1.00	1.00	1.00	1.00
*Few*	1.45 (1.23, 1.71)	1.34 (0.89, 2.02)	1.73 (1.20, 2.50)	0.97 (0.67, 1.41)	3.09 (1.79, 5.33)	1.49 (1.09, 2.04)
*Less than half*	2.35 (1.87, 2.95)	1.66 (1.00, 2.76)	2.25 (1.33, 3.81)	1.25 (0.74, 2.09)	8.40 (4.48, 15.74)	2.69 (1.58, 4.56)
*About half or more*	3.97 (3.22, 4.88)	2.57 (1.64, 4.02)	4.03 (2.46, 6.63)	3.17 (2.08, 4.85)	8.26 (4.58, 14.89)	12.37 (5.99, 25.55)
**Parents smoke**						
*No*	1.00	1.00	1.00	1.00	1.00	1.00
*Yes*	1.67 (1.47, 1.89)	2.21 (1.65, 2.96)	1.40 (1.05, 1.87)	1.28 (0.97, 1.68)	2.21 (1.65, 2.96)	1.54 (1.16, 2.04)

Odds ratios (ever smoker: never smoker) adjusted for all above listed variables (and country for the combined analysis).

Having increasingly more friends and family who smoke was strongly associated with being more likely to smoke. This trend was significant overall and in each country-specific analysis. The associations were especially strong in the Czech Republic and in Sweden. Parent smoking was also significantly associated with ever smoking (Table 2).

In all countries, the primary reason women reported for starting to smoke was because their friends smoked (62.3%). The second most frequent reason reported was to make them look 'more cool' (25.5%). Only 6.1% of participants reported starting smoking because a family member smoked (Table 3). Some common responses listed under 'Other' were curiosity, rebellion, because other people were smoking, or because they had personal problems.

**Table 3:** Reasons reported for smoking initiation among ever smokers, weighted.*

	All (n = 2378) %	France (n = 467) %	Ireland (n = 519) %	Italy (n = 474) %	Czech Republic (n = 429) %	Sweden (n = 489) %	χ^2^ p-value
*My friends smoked*	62.3	67.7	65.4	58.6	61.3	58.3	0.0152
*Smoking made me look more cool*	25.5	14.2	35.4	19.4	28.1	27.6	<0.0001
*My friends approved of my smoking*	6.9	5.5	16.3	0.9	10.3	0.6	<0.0001
*It helped me manage my stress*	6.8	8.9	8.1	7.2	7.6	2.6	0.0021
*My family members smoked*	6.1	6.1	11.1	6.4	4.6	1.7	<0.0001
*I was not worried about health effects*	5.9	0.4	21.1	0.2	0.9	3.0	<0.0001
*I believed I could quit whenever I wanted to*	5.6	0.7	18.2	0.2	4.1	1.8	<0.0001
*Tobacco advertising was attractive to me*	1.7	1.4	4.6	0.1	0.4	1.0	<0.0001
*Weight concerns*	1.5	1.0	5.0	0.4	0.4	0.2	<0.0001
*I was less depressed when I smoked*	1.5	1.5	1.7	2.4	1.1	0.8	0.4108
*My parents did not mind that I smoked*	0.6	0.4	0.5	0.6	0.5	1.1	0.7187
*Other*	15.6	11.4	14.5	24.5	13.3	14.2	<0.0001

*Participants were allowed to list more than one reason for smoking initiation.

Average age at smoking initiation was 18.2 and ranged from 10 to 60 years old. Average age at smoking initiation was 17.1 in Sweden, 17.9 in Ireland, 18.1 in France, 18.6 in Italy, and 19.6 in the Czech Republic. Over 80% of women smokers had started smoking by the age of 20. Sweden had the highest percentage of women who started smoking at a very young age with 29.3% starting between 14 and 15 years of age and 12.0% starting before the age of 14. The Czech Republic had the lowest percentage of young initiators with 13.7% starting between the ages of 14 and 15 and 1.4% starting younger than 14 years old. The highest percentage of women began smoking between the ages of 16 and 17 in all countries, except Sweden (Table 4).

**Table 4:** Distribution of age at smoking initiation by country, weighted.

	All	France	Ireland	Italy	Czech Republic	Sweden
	(n = 2364)	(n = 461)	(n = 515)	(n = 472)	(n = 427)	(n = 489)
**Mean age at initiation**	18.2	18.1	17.9	18.6	19.6	17.1
(ANOVA p-value < 0.01)						

**Range and quartile distribution of age at initiation**						
*Maximum*	60	50	50	55	60	45
*75% quartile*	20	19	20	20	20	18
*Median*	17	17	17	18	18	16
*25% quartile*	15	16	15	15	16	14
*Minimum*	10	11	10	12	10	11

**Age at initiation**						
*<14*	6.8%	3.2%	9.0%	6.0%	1.4%	12.0%
*14-15*	20.7%	18.1%	20.2%	19.7%	13.7%	29.3%
*16-17*	24.7%	29.6%	23.2%	22.6%	25.9%	23.4%
*18-19*	20.4%	24.7%	21.5%	20.6%	20.6%	15.6%
*20-21*	13.3%	12.7%	13.0%	14.3%	18.2%	9.6%
*22-23*	4.0%	3.4%	3.7%	5.1%	3.9%	3.7%
*24-25*	3.7%	2.6%	4.6%	4.0%	4.8%	2.6%
*26-27*	1.0%	0.8%	0.7%	1.1%	1.9%	0.9%
*28-29*	0.8%	0.7%	1.0%	0.4%	0.9%	1.0%
*30+*	4.6%	4.2%	3.1%	6.2%	8.7%	2.0%

Overall, those who reported currently having lower income had a higher average age of smoking initiation. Average age at initiation did not vary significantly by education in the combined analysis, but did vary significantly in France and Sweden. Women in the younger age cohorts generally began smoking at a younger age than women in older age cohorts (Table 5). Linear regression analysis showed that, after controlling for reasons for starting smoking, age at initiation was significantly earlier among Swedish women (β -0.72, p = 0.02) than among French women, and significantly later among women from the Czech Republic (β 1.79, p < 0.0001) (Table 6). Women who started smoking because their friends smoked or to look 'cool' were more likely to start smoking at a younger age than those who did not cite these reasons. Women who smoked to manage stress or to feel less depressed were more likely to start at an older age (Table 6).

**Table 5:** Mean age at smoking initiation by income, education, and age, weighted.

	All (n = 2364)		France (n = 461)		Ireland (n = 515)		Italy (n = 472)		Czech Republic (n = 427)		Sweden (n = 489)	
**Mean age at initiation**	**n**	**mean**	**n**	**mean**	**n**	**mean**	**n**	**mean**	**n**	**mean**	**n**	**mean**

**by current income**												
*Well below the median*	241	19.3	49	18.5	41	20.3	88	18.7	28	20.9	35	19.1
*Below the median*	591	18.3	114	17.8	144	17.9	143	18.3	92	21.1	98	17.0
*Around the median*	647	18.1	111	18.4	144	17.6	113	18.3	175	18.9	104	17.0
*Above the median*	400	17.7	79	18.4	107	17.4	41	18.6	71	18.7	102	16.6
*Well above the median*	79	17.9	14	20.0	11	19.6	4	22.8	4	17.3	46	16.6
*Refused to answer*	406	18.1	94	17.3	68	17.8	83	19.3	57	20.2	104	17.0
(ANOVA p-value)	(p = 0.0025)		(p = 0.0945)		(p = 0.0101)		(p = 0.4711)		(p = 0.0077)		(p = 0.0566)	

**by age at last education**												
*<16*	288	18.8	21	20.9	89	17.9	104	19.7	19	21.2	55	17.3
*16-19*	1052	18.0	165	18.2	278	18.0	175	18.1	228	19.7	206	16.3
*20-25*	713	18.2	214	18.0	112	17.8	140	18.3	138	19.1	109	17.7
*>25*	229	18.3	19	17.6	30	18.3	50	18.8	35	20.3	95	17.6
*Missing*	82	17.3	42	17.2	6	14.3	3	19.4	7	18.9	24	17.5
(ANOVA p-value)	(p = 0.0913)		(p = 0.0162)		(p = 0.9321)		(p = 0.0807)		(p = 0.3701)		(p = 0.0071)	

by current age*												
*18-24*	184		30		50		30		37		37	
*25-34*	419	16.4	76	16.9	110	16.4	92	16.3	80	17.2	61	15.2
*35-44*	380	17.0	61	17.2	102	17.2	74	17.1	52	17.8	91	16.2
*45-54*	391	17.0	74	16.8	78	17.0	87	17.3	60	17.9	92	16.2
*55+*	743	17.6	179	17.7	129	17.6	136	17.9	122	18.5	177	16.9
(ANOVA p-value)	(p < 0.0001)		(p = 0.0002)		(p < 0.0001)		(p < 0.0001)		(p < 0.0001)		(p < 0.0001)	

*excluding those who initiated after the age of 24 (n = 245)

**Table 6:** Linear regression of factors associated with age at smoking initiation.

	*β *(95% CI)	p-value
**Intercept**	18.74 (18.20, 19.28)	<0.0001
**Country**		
France	-	
Ireland	0.22 (-0.38, 0.82)	0.47
Italy	0.54 (-0.06, 1.14)	0.08
Czech	1.79 (1.16, 2.41)	<0.0001
Sweden	-0.72 (-1.30, -0.14)	0.02
Reasons for initiation*		
My friends smoked	-1.02 (-1.42, -0.61)	<0.0001
Smoking made me look more cool	-1.69 (-2.15, -1.23)	<0.0001
My friends approved of my smoking	-0.36 (-1.11, 0.39)	0.35
It helped me manage my stress	2.67 (1.88, 3.45)	<0.0001
My family members smoked	-0.20 (-0.98, 0.58)	0.61
I was not worried about health effects	0.41 (-0.63, 1.45)	0.44
I believed I could quit whenever I wanted to	-0.65 (-1.69, 0.39)	0.22
Tobacco advertising was attractive to me	0.67 (-0.79, 2.14)	0.37
Weight concerns	0.45 (-1.08, 1.99)	0.56
I was less depressed when I smoked	3.88 (2.33, 5.42)	<0.0001
My parents did not mind that I smoked	-0.95 (-3.27, 1.37)	0.42

*Respondents could choose more than one reason for initiation. Those who gave each reason were compared with those who did not.

## Discussion

This study provides an overview of factors associated with women's smoking in five European countries at the beginning of a time of rapid change in tobacco policy. We found that friends exert a strong influence on a woman's decision to start smoking in all five countries, especially in younger women. This has been reported from previous studies based in Europe and in studies based in the United States, Iran, Thailand and Mexico [6-12,31-33]. We found that women in the Czech Republic and Sweden appeared to be more strongly influenced by friends and family smoking than in other countries.

We found that being divorced or separated is strongly associated with smoking, which has been reported previously [34]. The association between divorce or separation and ever smoking appears to be strongest in Ireland and Italy, where rates of divorce and separation were lowest in our study sample. Our results also showed that in France, women who had never married were more likely to smoke than those who were married. Chaix and colleagues found similar results when they conducted a nationwide survey in France of 12 948 men and women [35].

Overall, we did not find a strong association between socioeconomic measures and smoking. This is in contrast to other findings on education status, low socioeconomic status (SES), and smoking initiation from Europe and other parts of the world [14-16,36-41]. Our null findings could have partly been due the way the data were collected. Instead of asking for what level of education had been achieved, participants were asked how old they were when they completed their education. As individuals can complete different levels of education at different ages, this may have made our education groups more similar in terms of actual education level and biased the results toward the null. Also, since income can be a sensitive topic, there may have been reporting bias. The distribution of income levels was not as we expected; there was a disproportionate number of Italian women who reported having income well below the median and a disproportionate number of Czech women who reported having income around the median. This may be an indication that the way the question was asked made the responses subjective. There was a considerable amount of variation in the response rate between countries. Only 41.4% of women in Italy and 30.6% of women in the Czech Republic contacted who were eligible for participation agreed to participate. This may be another reason why the income distribution for these two countries was skewed. Our null findings on socio-economic status and smoking initiation could have also been due to generational differences between the younger and older women. In younger women, less education has been found to be associated with ever smoking; however, education has been found to be less predictive for older age groups. The generational shift varies in different European countries, with larger inequalities in Northern Europe, and this could have also diluted our overall results [42-47].

Alternatively, the null association between socioeconomic measures and ever smoking could in fact be accurate. Most studies investigating smoking initiation and socioeconomic factors look at populations that include both men and women. Women, however, may be less influenced by education and income status than men and more influenced by social factors such as friends and family. Many studies looking at SES and smoking status measure SES at the neighborhood level [14,15,36,38]. Using neighborhood level data, rather than individual level data, may account for societal factors that may have a strong influence on women. One study showed that higher levels of smoking in deprived areas can only be partially explained by individual SES [15]. Similarly, a study based in California found that the protective effects of individual SES may be reduced if an individual lives in a low SES neighborhood [36]. Further investigation may provide interesting insight into what specific aspects of SES influence smoking in women.

As has been seen elsewhere [6-12], friends smoking was the top reason given for smoking initiation. The second most common reason for initiation was to 'look cool'. Spijkerman and colleagues also found that smoking was associated with wanting to be cool, rebellious and attractive [48]. Watson and colleagues found that smoking was accepted as part of a 'cool' image [49]. Friends smoking and looking cool was especially important for the younger women in our study.

Over 80% of the smokers in our study started smoking by 20 years of age. Those who initiate smoking in adolescence are of particular concern. Adolescents are more susceptible to nicotine addiction and require a shorter duration of smoking and fewer cigarettes to become addicted [50]. Furthermore, the earlier one starts smoking, the greater the cancer risk in middle and old age [50,51]. Average age at smoking initiation varied by country, income, education, and age cohort. Schulze and Mons also found mean age of smoking initiation differed by education level in Germany, and they found that the gap was widening among women [52]. Consistent with our findings, La Vecchia et al. found that Italian women are initiating smoking at an earlier age than previous generations [53]. Differences in age at initiation by country may be reflected in inter-country variation in lung cancer mortality by age [3].

We found depression to be associated with later smoking initiation. Since median age of onset of mood disorders, including major depressive disorder, ranges from 29 to 43 globally [54], it is not surprising that women who initiated smoking at a later age cited depression as a factor. In our study sample, 7.3% of women who started smoking at age 29 and older cited depression as a reason for initiating smoking compared to 1.2% in women who started smoking younger than 29 (p < 0.0001, data not shown). There is already much evidence linking depression and smoking [55,56]; however, there is some debate as to the direction of the causality [57-61].

However, since depression was only evaluated by self-report, the definition of depression may have been used very broadly by the participants and it is difficult to make any specific clinical conclusions. It may be interesting for future studies to investigate if the relationship between depression and smoking differs by age at smoking initiation.

We found that individuals who stated they began smoking to reduce stress were more likely to have initiated at an older age. Other studies have found an association between stress and smoking [62-64]. Lloyd and Taylor found that the effect of former life stress predicts smoking independently of recent stress and that the effect of social stress on smoking is additive over time [65]. In other words, stress at a younger age may affect future smoking; therefore, women who initiated smoking at a later age were more likely to cite stress as a factor.

One major limitation of the study was the stratified sampling approach using available telephone numbers. This method was chosen to allow the study to reach a large sample of women that was proportionally representative of smoking rates in each age group. However, administering the survey via telephone prevented us from verifying self-reported data. The potential for recall bias on reported age of initiation may have affected the accuracy of results. This bias was not likely to be differential by age group and thus should not affect the overall picture of the results. Also, since no mobile phone numbers were included in the phone list, the study could have excluded a substantial number of women from our study who may have unknown differences than those who could be reached by home telephone. Level of income and/or education may have affected whether women had a landline or whether they relied solely on cellular phone use. Thus, the participants may not be an accurate representation of their respective countries.

Though the five countries included in our study were at different stages of the tobacco epidemic [27] factors associated with smoking initiation were very similar. The association between friends and family smoking was found in all countries, but was strongest in Sweden and Czech Republic, two countries with very different tobacco epidemics. Mean age of smoking initiation was youngest in Sweden, which is in the more advanced stages of the tobacco epidemic. Mean age of initiation was oldest in the Czech Republic, which is in an earlier stage of the tobacco epidemic.

As rates of smoking are still rising among women in much of Europe, there is much to be done to prevent smoking-attributable mortality rates from also rising. The recent changes in Europe's tobacco policy have shown promising signs of slowing the epidemic. In a cross-sectional study of 18 European countries, countries with more developed tobacco control policies had higher levels of smoking cessation than in countries with less developed tobacco policies [66]. In Ireland, Mullally *et al*. found a significant decline in cigarette consumption among bar workers and a significant drop in smoking prevalence in the general population one year after a nationwide ban on workplace smoking in March 2004 [67]. In Scotland, Fowkes *et al*. found that smoking quit rates increased in the three months prior to introduction of the Scottish smoke-free legislation in March 2006 [68].

## Conclusions

This study showed that among European women, friends are the primary factor influencing both ever smoking and smoking initiation, especially among young women. We also found that over 80% of European women begin smoking by the age of 20 and that average age of smoking initiation varies by country, income, and age cohort, with Swedish women most likely to start smoking at a young age. The results from this study may serve as a helpful baseline for future studies on the long-term effect of the recent European tobacco laws.

## Competing interests

This research was funded by the European Union, in the framework of the Public Health Programme.

## Authors' contributions

DLO analyzed the data and drafted the manuscript. JEH assisted in the design of the study and the statistical analysis. CD, SA, M Haglund, SSDM, EK, IS, ET, and ERG helped to draft the manuscript. M Hashibe conceived of the study, and participated in its design and coordination and helped to draft the manuscript. All authors read and approved the final manuscript.

## Pre-publication history

The pre-publication history for this paper can be accessed here:

http://www.biomedcentral.com/1471-2458/10/74/prepub
